# Emerging evidences for the opposite role of apolipoprotein C3 and apolipoprotein A5 in lipid metabolism and coronary artery disease

**DOI:** 10.1186/s12944-019-1166-5

**Published:** 2019-12-13

**Authors:** Wen Dai, Ziyu Zhang, Chun Yao, Shuiping Zhao

**Affiliations:** Department of Cardiology, The Second Xiangya Hospital, Central South University, No. 139, Middle Renmin Road, Changsha, 410011 China

**Keywords:** Apolipoprotein C3, Apolipoprotein A5, Coronary artery disease, Atherosclerosis, Remnant cholesterol

## Abstract

Apolipoprotein C3 (apoC3) and apolipoprotein A5 (apoA5), encoded by *APOA1/C3/A4/A5* gene cluster, are two critical regulators of plasma triglyceride (TG) metabolism. Deficiency of apoC3 or apoA5 led to significant decreased or increased plasma TG levels, respectively. Recent studies indicated apoC3 and apoA5 also played roles in plasma remnant cholesterol, high density lipoprotein (HDL) and hepatic TG metabolisms. Moreover, large scale population genetic studies indicated that loss of function mutations in *APOC3* and *APOA5* gene conferred decreased and increased risk of coronary artery disease (CAD), respectively. This manuscript mainly reviewed existing evidences suggesting the opposite role of apoC3 and apoA5 in lipid metabolism and CAD risk, and discussed the potential correlation between these two apolipoproteins.

## Introduction

Apolipoprotein C3 (apoC3) and apolipoprotein A5 (apoA5) are encoded by *APOA1/C3/A4/A5* gene clusters. Evidences from genetic, epidemiological studies and basic experiments have consistently demonstrated that apoC3 and apoA5 are critical modulators of plasma triglyceride (TG) metabolism [1, 2]. Deficiency of apoC3 or apoA5 led to significant decreased or increased plasma TG level in human and mice [1, 2]. In-depth mechanistic studies revealed apoC3 inhibited plasma TG hydrolysis, remnant lipoprotein uptake and promoted hepatic TG secretion, while apoA5 regulated plasma TG metabolisms in a completely opposite manner [1, 2]. Recent studies further revealed additional role of apoC3 and apoA5 in remnant cholesterol (RC), high density lipoprotein (HDL) and hepatic TG metabolism [1, 2]. Moreover, large scale population genetic studies indicated that loss of function mutations in *APOC3* and *APOA5* gene conferred decreased and increased risk of coronary artery disease (CAD) [3–8], respectively. Thus, apoC3 and apoA5 emerge as potential novel targets to reduce cardiovascular risk. This manuscript mainly reviewed the existing evidences suggesting the opposite role of apoC3 and apoA5 in lipid metabolism and CAD risk, and discussed potential correlation between these two apolipoproteins.

### Gene structure and expression regulation

Human *APOA1/C3/A4/A5* gene clusters are located on chromosome 11q23, where *APOC3* gene is approximately 35 kbp upstream from the *APOA5* gene locus [9]. Their sequences are evolutionarily conserved [10, 11]. Human *APOC3* gene regulatory regions contain a set of proximal promotor with four elements (− 283/+ 24) and distal enhancer with six elements (− 890/− 500) [9]. Earlier animal and cell culture studies established that *APOC3* enhancer acted as a common regulatory sequence to direct hepatic and intestinal *APOA1*, *APOC3*, and *APOA4* gene expression [9]. However, sufficient liver specific *APOA5* gene expression was obtained in vivo with a 26 kb DNA *XhoI*-fragment containing only the *APOA5* gene and thus lacking *APOC3* enhancer [10]. Gao et al. further confirmed the *APOC3* enhancer didn’t affect *APOA5* expression in transgenic mice [12]. Actually, two elements in *APOA5* promotor region have been found critical to direct its expression in human hepatic cell lines [13, 14].

Initiation of gene expression is executed by specific binding of transcription factors to gene regulatory elements, and molecules affecting this process can regulate corresponding gene expression. The concrete structure and regulation mechanisms of *APOC3* and *APOA5* gene expression have been reviewed elsewhere [9], and we will focus here on regulators that are shared by *APOC3* and *APOA5*. Indeed, several molecules have been implicated in the same direction regulation of *APOC3* and *APOA5* expression, including upregulation with hepatocyte nuclear factor 4-α (HNF4-α) [15, 16] and glucose [17, 18], and downregulation with AMP-activated protein kinase [15, 19], insulin [20–22] and tumor necrosis factor-α (TNF-α) [23, 24]. Noticeably, these substances, except for TNF-α, are all important components directly involved in glucose metabolism, suggesting *APOC3* and *APOA5* dysregulation may contribute to diabetic dyslipidemia. Opposite direction regulation was also found in that peroxisome proliferator-activated receptor-α (PPAR-α) and farnesoid X-activated receptor (FXR) promoted *APOA5* [13, 14] while inhibited *APOC3* expression [25, 26]. In contrast to *APOA5*, the human *APOC3* gene promoter doesn’t contain PPAR-α and FXR positive response elements. Actually, these two nuclear receptors acted indirectly by interfering the binding of other transcriptional factors, like HNF4-α, to specific elements of *APOC3*, thereby further inhibiting *APOC3* gene transcription [26, 27]. Thus, the plasma TG lowering effect of fibrates, one type of PPAR-α agonists, may be partly mediated by increasing the circulating concentration of apoA5 and/or decreasing apoC3 levels. Indeed, recent studies showed that both fenofibrates and omega-3 polyunsaturated fatty acids therapy significantly decreased plasma apoC3 levels in humans [28, 29].

### Plasma lipid metabolism

#### Lipoprotein distribution

Circulating apoC3 and apoA5 were mainly associated with triglyceride rich protein (TRL) and HDL [11, 30]. Studies showed either of apoC3 and apoA5 was exchangeable between TRL and HDL [31]. In normolipidemia state of human subjects, the majority of plasma apoC3 was bound to HDL [32]. On the contrary, in subjects with hypertriglyceridemia (HTG), apoC3 was mostly found on very low density lipoprotein (VLDL) [33]. With the concentration of TG in artificial TG emulsions increasing, a greater fraction of apoC3 shifted away from native plasma lipoproteins to artificial emulsions [33]. Glangeaud et al. [34] found during the lipoprotein lipase (LPL) mediated hydrolysis of VLDL, apoC3 redistributed from VLDL to HDL in vitro study, with the amount that was proportional to the magnitude of TG hydrolysis in VLDL, and apoC3 was subsequently transferred back to newly synthesized TG-enriched VLDL particles [11]. Similarly, Nelbach et al. [35] demonstrated apoA5 was predominantly associated with HDL in *APOA5* transgenic mice, which had TG-rare VLDL, but was rapidly and efficiently redistributed to TG-rich VLDL isolated from *APOA5* knockout mice upon incubation. Shu et al. [36] also reported that intravenous injection of apoA5-containing reconstituted HDL in *APOA5* knockout mice showed the identical exchange pattern of apoA5 between reconstituted HDL and VLDL, and apoA5 still remained associated with the TG-rich VLDL due to the disruption of VLDL hydrolysis.

These findings suggested that lipoprotein distributions of apoC3 and apoA5 were closely associated with the TG contents in TRL. The majority of apoC3 and apoA5 were in HDL when there was low TG levels in TRL. A large portion of apoC3 and apoA5 redistributed from HDL to TRL particles when TG amounts increased in TRL, and they gradually shuttled back to HDL with the processing of TRL hydrolysis. However, the biologic function and regulation mechanism of the exchange process have not been well elucidated.

#### Plasma TG

ApoC3 and apoA5 are critical determinants of plasma TG concentration as evidenced by genetic observations in humans. Loss of function mutations in human *APOC3* gene conferred a low plasma TG profile [3–5], while patients with *APOA5* deficiency mutation had extremely high plasma TG levels [37–39]. Abnormalities in apoC3 and apoA5 were associated with different forms of HTG, such as familial hyperchylomicronemia [38, 40], familial combined hyperlipidemia [41–43], and familial dysbetalipoproteniemia [44]. Interestingly, recent studies showed the existence of single glycosylation site at Threonine 74 of apoC3 protein, giving rise to four major proteoforms in plasma. The wild-type form which does not contain a glycan chain is commonly referred to apoC3_0a_. The rest other three all have a core glycan chain made of an O-linked disaccharide galactose linked to N-acetylgalactosamine. ApoC3_0b_ is the proteoform that just contain the glycan core, while apoC3_1_ and apoC3_2_ contain additional one and two sialic acid residues, respectively [45]. Moreover, four major proteoforms of apoC3 differentially correlated to fasting TG levels. It has been found that, using mass spectrometric immunoassay measurement, plasma apoC3_0a_, apoC3_0b_, and apoC3_1_ had positive while apoC3_2_ had negative relationship with fasting plasma TG [45], suggesting that analysis of individual isoforms of apoC3 could provide more comprehensive information than total plasma apoC3 concentration only.

Consistently, *APOC3* knockout mice had decreased TG concentration (− 30%) compared to wild littermates, while *APOC3* transgenic mice showed increased serum TG level (+ 200% to 2000%) [46, 47]. On the other hand, *APOA5* knockouts had an increase (+ 400%) in TG levels whereas *APOA5* transgenic mice displayed significantly reduced (− 70%) in this lipid parameter [48].

**Fig. 1:**
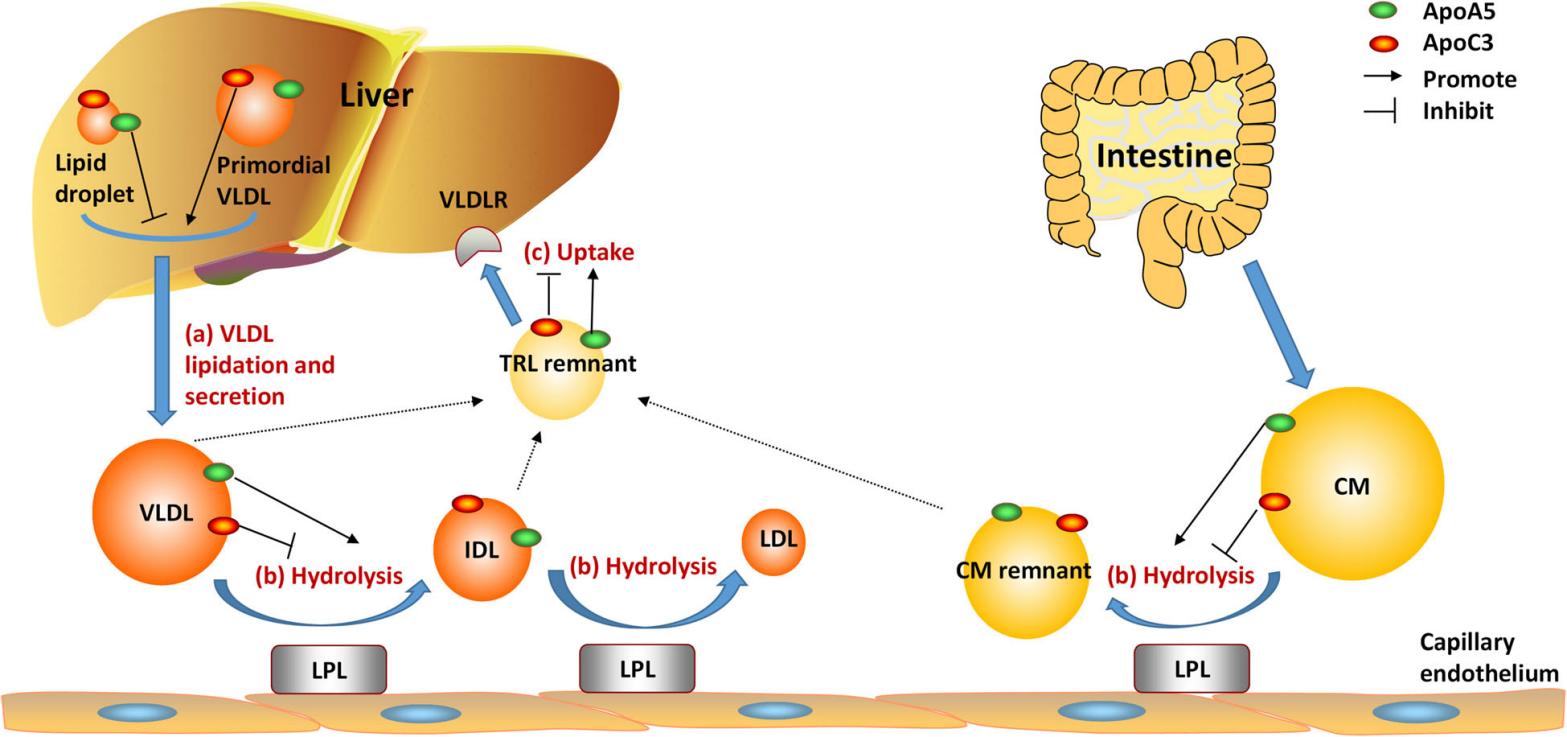
The opposite role of apoC3 and apoA5 in TRL metabolism. ApoC3 and apoA5 regulated TRL metabolism through multiple pathways: (**a**). hepatic VLDL lipidation and secretion; (**b**). LPL mediated TRL hydrolysis; (**c**). TRL remnant clearance via hepatic uptake. ApoC3 inhibited LPL mediated TRL hydrolysis, circulating TRL remnant clearance and promoted hepatic VLDL-TG secretion. Conversely, apoA5 accelerated TRL hydrolysis, TRL remnant uptake by liver while inhibited hepatic VLDL-TG secretion. ApoC3, apolipoprotein C3; apoA5, apolipoprotein A5; TRL, triglyceride rich lipoprotein; VLDL, very low density lipoprotein; LPL, lipoprotein lipase; IDL, intermediate density lipoprotein; LDL, low density lipoprotein; CM, chylomicron

In-depth mechanistic studies revealed that apoC3 and apoA5 regulated plasma TG levels through multiple pathways. ApoC3 inhibited LPL mediated TRL hydrolysis, circulating TRL remnant clearance and promoted hepatic TG secretion. Interestingly, apoA5 regulated plasma TG metabolism in a completely opposite manner. Namely, apoA5 accelerated TRL hydrolysis, TRL remnants uptake by liver while inhibited hepatic TG secretion [1, 49] (Fig. 1).

#### Plasma RC

RC is defined as the total cholesterol contents of TRL, including VLDL and intermediate-density lipoproteins (IDL) in the fasting state, and VLDL, IDL, and chylomicron remnants in the non-fasting state. Growing evidence indicated that RC is an independent causal risk factor of ischemic heart disease [50, 51]. Moreover, elevated RC levels were associated with increased all-cause mortality in patients with ischemic heart disease [52].

Since apoC3 and apoA5 regulated TRL metabolisms, it is not unexpectedly to find that *APOC3* and *APOA5* gene variants were associated with RC levels. In a meta-analyses of 137,895 individuals, RC was 43% lower in *APOC3* loss-of-function heterozygotes versus noncarriers [53]. Contrarily, genotype combinations of common variants of *APOA5* (c.-1131 T > C, S19 W, and c.*31C > T) associated with increases in RC of up to 56% [6]. Thus, targeting apoC3 or apoA5 seems to be a potential approach to reduce plasma RC levels, which could be testified in future trials.

#### HDL

HDL exerts various athero-protective properties, including mediating cholesterol efflux, protecting vascular endothelium, anti-inflammatory and anti-apoptotic effects [54]. HDL with deficiencies in these properties is referred to as dysfunctional HDL, which in turn contributes to the progression of CAD. Human observation studies indicated these properties were defective under pathological disorders. For example, impaired cholesterol efflux capacity was found with HDL from uremic patients [55]. Riwanto et al. [56] found HDL from CAD patients didn’t activate endothelial anti-apoptotic pathways, but rather stimulates potential endothelial pro-apoptotic pathways. By spectrometry and biochemical analyses, studies further indicated impaired HDL function correlated closely to the alteration in its proteome composition [54, 55, 57], among which changes of apoC3 and apoA5 gained lots of attentions.

Riwanto et al. found that there were significant higher apoC3 in HDL particle from CAD patients compared to healthy controls. Besides, using antibody neutralizing apoC3 in these HDL improved HDL mediated anti-endothelial apoptosis function [56]. Cho KH showed increasing apoC3 content in artificial reconstituted HDL reduced its lecithin cholesterol acyltransferase (LCAT) activation ability [58]. Interestingly, Luo M et al. demonstrated ApoC3 contents in HDL were negatively associated with HDL-mediated cholesterol efflux capacity [59], however, underlying mechanism are unknown. By contrast, adenovirus-mediated overexpression of *APOA5* in mice led to increased apoA5 in HDL, associated with increased cholesterol efflux capacity [60]. Reconstituted HDL synthesized with more apoA5 had larger particle size, more lipid content and better antioxidant capacity against LDL in vitro [61].

The definite role of apoC3 and apoA5 in HDL function need to be further examined. It’s been reported that apoC3 in HDL can bind to scavenger receptor B1 (SR-B1) [62], with uncharacterized structure domain. SR-B1 is known as an important element in cholesterol reverse transportation partly for facilitating the selectively uptake of cholesterol esters from HDL by the liver [62]. Whether this interaction of apoC3 with SR-B1 would influence cholesterol reverse transportation are undetermined.

### Hepatic VLDL secretion

One of the major functions of the liver is to synthesize and secrete VLDL. VLDL is composed of a core of neutral lipids, mostly TG, and several apolipoproteins [63]. Of which, apolipoprotein B100 (apoB100) is the most important and provides structural stability to the VLDL particle. There are two steps for the biogenesis of VLDL. Initially, the formation of VLDL starts with the synthesis of apoB100 in the endoplasmic reticulum (ER). The nascent apoB100 is then partially lapidated to form a lipid-poor primordial VLDL particle, which is facilitated by microsomal triglyceride transfer protein (MTP). In the second step of VLDL formation, the primordial VLDL particle fuses with triglyceride-rich particles to form mature TG-rich VLDL [63]. Growing evidences have indicated apoC3 and apoA5 regulated the VLDL lipidation and affected the hepatic TG content [1, 49] (Fig. 1).

Data from cell culture, animal experiments and human studies confirmed that apoA5 inhibited VLDL-TG secretion and promoted the storage of TG in cytosolic lipid droplet. McA-RH7777 cells stably transfected with human *APOA5* secrete VLDLs that were smaller than those from control cells, but had greater cell TG level and larger lipid droplets [64, 65]. By contrast, Ress et al. [66] reported that knockdown of *APOA5* in HepG2 cells led to a decrease in cellular TG content. Livers from *APOA5* transgenic mice had increased hepatic TG level compared with wild littermates [67]. Qin et al. [68] found patients with nonalcoholic fatty liver disease (NAFLD) have elevated *APOA5* expression compared to healthy controls. However, there’re still some puzzlements needed to be further elucidated. Primarily, whereby does a portion of apoA5 escape secretion pathway into blood and become associated with cytosolic lipid droplets? Additionally, how does apoA5 promote hepatic TG storage in lipid droplet (LD) instead of secretion in the form of VLDL.

Conversely, in vivo and in vitro studies have shown apoC3 has a stimulatory effect on VLDL lipidation. Feeding *APOC3* knockout mice with a high fat diet for two weeks failed to stimulate VLDL-TG production, while reconstitution of *APOC3* expression using adenovirus encoding human apoC3 resulted in robust production of VLDL-TG [69]. The stimulatory effect of human apoC3 on the lipidation of VLDL was recapitulated in McA-RH7777 cells under lipid-rich condition [70]. Furthermore, site directed mutation of residues in lipid binding domain (K58E) of apoC3 abolished this stimulatory effect [69]. These findings were isupported in humans by the observations that two SNPs of *APOC3* (C-482 T, T-455C), leading to decreased *APOC3* expression, were correlated with increased hepatic TG level and higher prevalence of NAFLD in Asian indian population [71].

The subcellular location of apoA5 and apoC3 regulating VLDL lipidation are proposed to be the ER compartment. Gao et al. hypothesized that apoA5 may facilitate the ER-luminal LDs budding outward to form cytosolic LD and hence reduce the TG assembled into VLDL particles [65]. Qin et al. found apoC3 promoted the fusion of ER-luminal LD with VLDL particles during VLDL lipidation [69]. In-depth studies focusing on the molecular basis underlying the effect of apoA5 and apoC3 on VLDL lipidation and LD metabolism are needed, which will provide novel understanding of hepatic TG homeostasis.

### Association with CAD

CAD has become a major cause of death worldwide. Low-density lipoprotein cholesterol (LDL-C) is well-acknowledged as playing a crucial role in the pathogenesis of CAD, and lowering of plasma LDL-C results in significant reduction in the morbidity and mortality of CAD [72, 73]. However, it has been reported that many individuals still suffered CAD despite achieving therapeutic goal for LDL-C levels [74, 75]. Therefore, efforts are on to identify other modifiable risk factors to further reduce the risk of CAD. Population genetic data is free of confounding and reverse causation, and is thus recognized as an important way to identify novel potential risk factors of CAD.

Interestingly, it has been demonstrated that genetically reduced plasma apoC3 levels were associated with decreased risk of CAD in humans [3–5]. A nonsense mutation of *APOC3* gene, R19X, was associated with a 50% reduction of circulating apoC3 levels [5]. More importantly, carriers of the rare variant R19X had lower incidence of coronary artery calcification and lower Framingham 10 year CAD risk [5]. The cardioprotective effect of R19X and other three rare variants, two splice site mutations (IVS2 + 1G → A; IVS3 + 1G → T) and one missense mutation (A43T) in *APOC3* gene, was recently confirmed in two large-scale studies [3, 4]. In a study as a part of the Exome Sequencing Project of the National Heart, Lung, and Blood Institute [3], approximately 1 in 150 participants was a heterozygous carrier of at least one of these four mutations, and circulating levels of *APOC3* in carriers were 46% lower than levels in noncarriers. The risk of CAD among 498 carriers of any rare *APOC3* mutation was 40% lower than the risk among 110, 472 noncarriers. Consistently, in a cohort of 75,725 participants, the cumulative incidences of ischemic vascular disease and ischemic heart disease were reduced in heterozygotes for loss-of-function mutations in *APOC3* (R19X or A43T or IVS2 + 1G → A) as compared with noncarriers, with corresponding risk reductions of 41% and 36% [4]. Noticeably, it has been reported that there was also a trend for fewer major adverse cardiovascular disease events in patients with higher apoC3_2_ proteoform, while these associations were not detected for the other apoC3 proteoforms, suggesting apoC3_2_ is more like a loss-of-function proteoform [45].

Contrarily, *APOA5* variants leading to decreased apoA5 levels were associated with increased CAD risk [6–8, 76]. The association between –1131 T > C promoter polymorphism of *APOA5* gene and risk of CAD has been shown in a large meta-analysis. The odds ratio for CAD was 1.18 per C vs. T allele [8]. Furthermore, several independent studies have consistently indicated that *APOA5* variants were significantly associated with the risk of myocardial infarction (MI). Raffaele De Caterina et al. found strong association of the *APOA5* -1131 T > C gene variant and early-onset acute MI [77]. Jorgensen AB et al. further showed genetic variation in the *APOA5* gene (c.-1131 T. C, S19 W, and c.*31C. T) associated with an 87% increase in MI risk [6]. Do R et al. sequenced the exons of *APOA5* in 6721 subjects with MI and 6711 controls. 46 unique non-synonymous or splice-site single nucleotide variants or indel frameshifts with allele frequency < 1% were identified. Moreover, carriers of these rare mutations in *APOA5* gene (1.4% of cases versus 0.6% of controls) were at 2.2-fold increased risk for MI compared to controls [7].

Furthermore, it has been suggested the effects of apoC3 and apoA5 on CAD risk are partially mediated by changes in plasma RC levels. Wulff AB et al. found RC mediated 37% of the observed 41% lower risk of ischemic vascular disease and 54% of the observed 36% lower risk of ischemic heart disease in *APOC3* loss-of-function heterozygotes versus noncarriers [53]. However, *APOA5* gene variants (c.-1131 T. C, S19 W, and c.*31C. T) leading to genetically increased RC associated with an increased risk of MI [6]. On the other hand, *APOA5* gene variants (c.-1131 T. C, S19 W, and c.*31C. T) associated with increases in RC of up to 56%, and with a corresponding odds ratio for MI of 1.87 [6].

### Potential correlation between apoC3 and apoA5

Since apoC3 and apoA5 regulate lipid metabolism and associate with CAD risk in an opposite manner, it is reasonable to wonder whether they function independently or cooperately. Some findings from genetic mice suggested a close relationship between these two proteins though there is no current evidence showing the direct interaction between them. Pennacchio et al. [48] demonstrated that *APOA5* transgenic and knockout mice have obviously decreased and increased hepatic apoC3 protein level, respectively, while with no significant changes found in apoC3 mRNA abundance. Indeed, the apoC3 protein amounts in liver were increased 90% in *APOA5* knockout mice and decreased 40% in *APOA5* transgenics compared to wild type littermates. Similarly, declined serum apoC3 level was observed after adenovirus-mediated overexpression of human *APOA5* in mice [60]. These findings implied that apoC3 may affected apoA5 at transcriptional levels, and vice versa. However, the underlying mechanisms are unknown.

## Conclusion

Considerable evidences showed apoC3 and apoA5 played important and opposite roles in lipid metabolism and CAD risk. Targeting apoC3 and apoA5 may be an intriguing therapy for lipid management and cardiovascular protection, which should be testified in future clinical trials.

## Data Availability

Not applicable.

## Abbreviations

apoA5: Apolipoprotein A5
apoB100: Apolipoprotein B100
ApoC3: Apolipoprotein C3
CAD: Coronary artery disease
ER: Endoplasmic reticulum
FXR: Farnesoid X-activated receptor
HDL: High density lipoprotein
HNF4-α: Hepatocyte nuclear factor 4-α
HTG: Hypertriglyceridemia
LCAT: Lecithin cholesterol acyltransferase
LD: Lipid droplet
LDL-C: Low-density lipoprotein cholesterol
LPL: Lipoprotein lipase
MTP: Microsomal triglyceride transfer protein
NAFLD: Nonalcoholic fatty liver disease
PPAR-α: Peroxisome proliferator-activated receptor-α
RC: Remnant cholesterol
SR-B1: Scavenger receptor B1
TG: Triglyceride
TNF-α: Tumor necrosis factor-α
TRL: Triglyceride rich lipoprotein
VLDL: Very low density lipoprotein
